# Knowledge, attitude and practice of breastfeeding in the north of Jordan: a cross-sectional study

**DOI:** 10.1186/1746-4358-1-17

**Published:** 2006-09-23

**Authors:** Mohammad Khassawneh, Yousef Khader, Zouhair Amarin, Ahmad Alkafajei

**Affiliations:** 1 Department of Paediatrics, Jordan University of Science and Technology, Irbid, Jordan; 2 Department of Public Health, Community Medicine and Family Medicine, Jordan University of Science and Technology, Irbid, Jordan; 3 Department of Obstetrics and Gynecology, Jordan University of Science and Technology, Irbid, Jordan

## Abstract

**Background:**

In Jordan, as in neighboring countries in the Middle East, higher education and higher employment rates in recent years among women have had an impact on traditionally based infant feeding. The objective of this study was to evaluate practice, knowledge and attitude to breastfeeding and to assess factors associated with breastfeeding among women in the north of Jordan.

**Methods:**

A cross sectional study was carried out between 15 July 2003 and 15 August 2003. A total of 344 women with children aged between 6 months and 3 years from five different villages in the north of Jordan were randomly selected and interviewed. Information regarding participants' demographics, infant feeding in first six months of life, knowledge and attitude towards breastfeeding was collected.

**Results:**

Full breastfeeding was reported by 58.3%, mixed feeding was reported by 30.3% and infant formula feeding was reported by 11.4%. Almost one third of the full breastfeeding group did so for 6–12 months, and almost two thirds did continue breastfeeding for more than one year. Employed women were more likely not to practice full breastfeeding compared to unemployed women (odds ratio 3.34, 95% CI 1.60, 6.98), and women who had caesarian delivery were more likely not to practice full breastfeeding compared to those who had vaginal delivery (odds ratio 2.36, 95% CI 1.17, 4.78). Jordanian women had a positive attitude but work place and short maternity leaves had a negative impact on breastfeeding.

**Conclusion:**

This study showed that a high proportion of Jordanian women did breastfeed for more than one year. However, working women and those who deliver by caesarean section were less likely to breastfeed. It is speculated that adopting facilitatory measures at hospitals and work place could increase the rate of full breastfeeding.

## Background

Breastfeeding rates vary in different countries. Little is known about breastfeeding and barriers women experience in the Middle East [1-4]. In addition, information is scant on how organizations can best promote breastfeeding in Jordan, a small economically disadvantaged developing country in southwestern Asia. To improve rates of full breastfeeding, specific information about the beliefs and practices that influence this outcome is needed. The current study was designed to evaluate practice, knowledge and attitude to breastfeeding and to assess factors associated with breastfeeding among women in the north of Jordan.

## Methods

A cross-sectional study was conducted between 15 July 2003 and 15 August 2003 among women residing in the north of Jordan. Considering the population size, geographic location, and the number of families, according to the National Department of Statistics, the study population consisted of 20 clusters. The study sampled 5 clusters out of these 20 (i.e. one fourth of the total number of clusters). The included clusters represented the whole 20 clusters of the Irbid Governorate, which are mainly semi-urban communities. The 5 selected clusters were chosen using systematic sampling technique. The proportion of ever-married women in the age group 15–49, as estimated by the Ministry of Health in 2000, was 55% [5]. For a sample size of 344 married women who had at least one child aged between 6 months and 3 years, the calculated power exceeded 80%, with 95% confidence that the true proportion of mothers that reported full breastfeeding is within ± 10% of the expected 50% proportion. Sample size was calculated using EPI-Info, 2002.

It was calculated that 625 households were needed to interview 344 married women. Having assumed that in every two households with married women one would have a targeted child, the number of households to be visited was doubled to 1250. Using systematic sampling technique (every 5^th ^cluster), primary sampling units of 5 clusters were selected. The households to be visited in each cluster were calculated using the "proportionate to size" method.

The data were collected by trained fourth year medical students from the Jordan University for Science and Technology. They were instructed on interview techniques and were familiar with the geography of these clusters. Oral consents were obtained from all participants and all approached women agreed to participate. The study was approved by the Scientific Research Board of the Jordan University of Science and Technology.

A structured questionnaire with 32 items was used. It covered demographic variables that included mother's age, mother's education, father's education, mother's employment status, total family income, family's size, mode of delivery, gender of last child, and history of neonatal hospitalization. Breastfeeding was divided into three types: full breastfeeding which included exclusive and almost exclusive breastfeeding for a duration of 6 months (exclusive breastfeeding when no other liquid or solid from any other source entered the infant's mouth, while almost exclusive breastfeeding when occasional tastes of other liquids, traditional foods, vitamins, medicines were allowed), mixed breastfeeding when infants received both artificial formula and breastfeeding, and exclusive bottlefeeding when infants received only artificial formula after 2 weeks of life. All mothers were asked questions regarding their main reasons for either breastfeeding or bottlefeeding.

Questions with five-point Likert rating scale, from strongly disagree to strongly agree were used to assess women's knowledge and attitude to breastfeeding. The original Likert rating scale was converted to 100% scale by multiplying the corresponding coefficients (i.e. 1, 2, 3, 4, and 5) by 20. A higher score indicated a higher participant's agreement with the item tested. Items testing knowledge included recommended breastfeeding duration, its benefit in decreasing the risk of acquiring diarrhea and its role in contraception. Questions that evaluated mothers' attitude included mother's comfort with breastfeeding, cost, effect on care of other family members and effect on marital relationship. Items that tested community's attitude toward breastfeeding included feeling shy of breastfeeding in public places, role of community nurses and medical staff in encouraging breastfeeding, duration of maternity leave and facilitation of breastfeeding at work places.

The Statistical Package for Social Sciences software (SPSS, version 11.5, Chicago. Inc) and Microsoft Office Excel 2003 were used for data processing and statistical analysis. Variables were described using frequency distribution for categorical variables and mean and standard deviation for continuous variables. The distribution of infant feeding practices by independent variables was tested using the Chi-square test. Factors associated with not practicing full breastfeeding were identified using univariate and multivariate logistic regression. Crude odds ratios and adjusted odds ratios and their 95% confidence intervals were reported. A p-value of less than 0.05 was considered significant.

## Results

### Participant characteristics

The age of the participants ranged between 17 and 45 years, with a mean age of 34.7 ± 5.1 years. The average total family income was 270.7 ± 128.9 Jordan Dinars/month (JD = US $1.42). About 60% of women were housewives, and 51.3% had a high school degree or more. A total of 195 (57.2%) women had 3 or more children (Table 1).

**Table 1:** Participants socio-demographic characteristics and type of delivery (n = 344).

	n	%
Age (year)		
<30	115	33.4
≥30	229	66.6
		
Education completed		
Less than high school	167	48.7
High school or higher	176	51.3
		
Paid employment		
Housewife	207	60.3
Employed	136	39.7
		
Income (Jordanian Dinar/month)		
<300	229	66.8
≥300	114	33.2
		
Number of children		
<3	146	42.8
≥3	195	57.2
		
Type of birth		
Vaginal	296	86.5
Cesarean section	46	13.5
		
Neonatal hospitalization		
Yes	44	12.9
No	296	87.1

### Type of feeding by socio-demographic and maternal characteristics

Of the 344 interviewed mothers, 58.3% were full breastfeeders, 30.3% were mixed breastfeeders, and 11.4% practiced infant formula feeding. Of the full breastfeeding group 32% did so for 6–12 months, and 68% of them did continue breastfeeding for more than one year. The types of feeding by socio-demographic and maternal characteristics are shown in Table 2. The type of feeding was independent of mother's age, father's education level, child's gender, and history of neonatal hospitalization. However, the type of feeding was associated with mothers' level of education and family income. Women with higher education and higher income were less likely to breastfeed. Similarly employed mothers, mothers with lower number of children and those delivered by caesarian were less likely to fully breastfeed.

**Table 2:** Type of feeding by socio-demographic and maternal characteristics.

	Type of feeding			p-value
	Breastfeeding (N = 200) n (%)*	Mixed (N = 94) n (%)	Infant Formula (N = 39) n (%)	
Age				0.146
<30	74 (64.3)	27 (23.5)	14 (12.2)	
≥30	126 (55.3)	77 (33.8)	25 (11.0)	
				
Mother education completed				0.024**
Less than high school	109 (65.7)	42 (25.3)	15 (9.0)	
More than high school	90 (51.1)	62 (35.2)	24 (13.6)	
				
Father education completed				0.293
Less than high school	115 (62.2)	51 (27.6)	19 (10.3)	
More than high school	85 (53.8)	53 (33.5)	20 (12.7)	
				
Income JD/month				0.039**
<300	138 (60.5)	68 (29.8)	22 (9.6)	
≥300	62 (54.4)	36 (31.6)	16 (14.0)	
				
Mothers' occupation completed				0.0001**
Housewife	139 (67.5)	49 (23.8)	18 (8.7)	
Employed	60 (44.1)	55 (40.4)	21 (15.4)	
				
Number of children				0.044**
<3	89 (61.0)	35 (24.0)	22 (15.1)	
≥3	110 (56.7)	67 (34.5)	17 (8.8)	
				
Gender of baby				0.685
Male	119 (60.1)	56 (28.3)	23 (11.6)	
Female	81 (56.3)	47 (32.6)	16 (11.1)	
				
Type of birth				0.001**
Vaginal	182 (61.5)	87 (29.4)	27 (9.1)	
Cesarean section	17 (37.0)	17 (37.0)	12 (26.1)	
				
Neonatal hospitalization				0.078
Yes	19 (43.2)	17 (38.6)	8 (18.2)	
No	179 (60.5)	86 (29.1)	31 (10.5)	

* Row percentage

** Statistically significant finding

**Table 3:** Factors associated with not full breastfeeding using logistic regression.

	Unadjusted OR (95% CI)	Adjusted OR (95% CI)
Mother's education		
≤ High school	1	1
> High school	1.83 (1.18, 2.83)*	1.08 (0.51, 2.30)
		
Employment		
No	1	1
Yes	2.63 (1.68, 4.11)*	3.34 (1.60, 6.98)*
		
Income		
<300	1	1
≥300	0.78 (0.49,1.22)	1.76 (0.87, 3.57)
		
Number of children		
<3	1	1
≥3	1.19 (0.77, 1.85)	1.58 (0.90, 2.76)
		
Type of delivery		
Vaginal	1	1
Caesarian	2.72 (1.43, 5.18)*	2.36 (1.17, 4.78)*

*Statistically significant finding

Seventy seven percent of all women in this study reported that "Breastfeeding is better for my baby's health" as the main reason for practicing full breastfeeding. While 37% of all women reported that mothers' employment being the main reason for bottlefeeding, and 33% thought that having insufficient breast milk was the main reason for that.

### Multivariate analysis of factors associated with not being a full breastfeeder

Factors that were associated with not being a full breastfeeder were entered into a logistic regression model. Employed women were more likely not to practice full breastfeeding compared with unemployed women (odds ratio 3.34, 95% CI 1.60, 6.98) and women who had caesarean delivery were more likely not to practice full breastfeeding compared to these with vaginal birth (odds ratio 2.36, 95% CI 1.17, 4.78).

### Knowledge and attitude to breastfeeding

Regarding women's knowledge, on a scale of 100, "breastfeeding for three months is considered long enough" scored 37, indicating disagreement with this item. Whereas most women agreed with the statement that breastfeeding is a good contraceptive method (score = 68) and that breastfeeding decreased diarrhea (score = 84).

Regarding the community's attitude to breastfeeding, on a scale of 100, the average score for answers are shown in Table 4. Mothers were not satisfied with the length of the maternity leave and the workplace facilities for breastfeeding. Jordanian women's positive attitude was reflected in their thinking that breastfeeding was easier than feeding infant formula and it was less expensive than feeding infant formula.

**Table 4:** Mothers' knowledge and attitudes to infant feeding.

Item	Average score*
*Knowledge*	
Three months of breastfeeding is long enough	37
Breastfeeding is a good contraceptive method	68
Breastfeeding decreases diarrhea	84
*Attitude*	
Breastfeeding being easier than feeding infant formula	84
It is not difficult for breastfeeding mother to care for family	71
Breastfeeding has no negative effect on marital relationship	63
Breastfeeding is a good way to decrease family expenses	81
Feeding infant formula keep the body well shaped and prevent over-weight	21
Community encourages breastfeeding over feeding infant formula	32
Doctors and nurses encourage breastfeeding	74
Maternity leave of 3 months is enough to successful breastfeeding	58
Work places provide designated areas for breastfeeding	30

* On 100 point scale, a higher score indicated a higher participant's agreement with the item tested.

## Discussion

This cross-sectional study demonstrated an initiation rate of breastfeeding of 88.6%. Over 58% were full and over 30% were mixed breastfeeders. These rates are higher than those reported in the USA for the same ages [6] but less than those reported in other Middle Eastern countries [2]. This may reflect a difference in the socio-economic and cultural status. In Jordan less-educated women were more likely to breastfeed than women with higher education levels. In contrast, education had a positive effect on breastfeeding in Western communities and developed countries [7,8].

The current study showed that factors associated with not practicing full breastfeeding were mothers' working status and delivery by caesarean section, where mothers rarely care for their babies in the first 2 days post-operatively. In Jordan there are only 3 hospitals that are accredited as being Baby Friendly. In contrast, in Lebanon, a Middle Eastern country with a similar population size and economic status, there are 21 such hospitals [9]. Hospitals and work places without facilities for breastfeeding can be detrimental for breastfeeding. Cohen and Mrtek showed that women employed by businesses that are "breastfeeding friendly" were able to maintain a breastfeeding regimen for at least six months at rates comparable to the rate of women who are not employed outside home [10].

Governmental policies regarding longer leave for new mothers and child care centers inside large institutions should be considered. The participants in this study were not satisfied with the length of their leave and they did not think that workplaces provided the right environment for successful breastfeeding.

Breastfeeding should be linked to public health education for the whole community. In an American survey, Li et al. [11] showed that the overall population approved breastfeeding in public places and that establishing work place breastfeeding policy and lactation rooms were the most acceptable modes for promoting breastfeeding. The participants in our study felt shy to breastfeed in public places. Consideration should be given to providing privacy to nursing mothers in both working and public places.

Almost 77% of the participants reported that the main reason for breastfeeding was "better for child's health". Media programs that emphasize the advantages of breastfeeding over infant formula feeding are probably the key point in spreading this practice. One major cause of infant formula feeding in the current study was related to mothers' concern that their milk was not sufficient (33%). Studies throughout the world have identified that concern about milk supply is the most common reason women give for stopping breastfeeding [12,13]. Education for women regarding time needed for colostrum to change to transitional milk and education regarding ways of successful breastfeeding can be valuable in decreasing concern about milk supply.

Jordanian women reported that the community and health care workers encouraged breastfeeding, but improvement in rights and education among Jordanian women, where only 5% are illiterate, have probably had an effect on feeling shy about breastfeeding in public places. This is different from the past in Jordan, where it was common to see mothers breastfeed in public places. Nowadays seeing this is considered unusual. We speculate that advertising infant formula may have contributed to the decrease in breastfeeding in public places.

A limitation of our study was its cross sectional design. For inclusion in this study, limiting the child's age to a maximum of 3 years would have diminished the risk of recall bias. Further cohort studies are needed to explore the effect of socioeconomic development on children health and nutrition.

## Conclusion

This study showed that a high proportion of Jordanian women did breastfeed for more than one year. However, working women and those who deliver by caesarean section were less likely to breastfeed. It is speculated that having Baby Friendly hospitals and work places would probably be steps in the right direction.

## Competing interests

The author(s) declare that they have no competing interests.

## Authors' contributions

MK: drafting the manuscript, revising it critically for important intellectual content and interpretation of data. YK: analysis and interpretation of data. ZA: drafting the manuscript, revising it critically for important intellectual content and interpretation of data. AA: supervision of the fieldwork, analysis and interpretation of data.
